# Novel Ergonomic Postural Assessment Method (NERPA) Using Product-Process Computer Aided Engineering for Ergonomic Workplace Design

**DOI:** 10.1371/journal.pone.0072703

**Published:** 2013-08-16

**Authors:** Alberto Sanchez-Lite, Manuel Garcia, Rosario Domingo, Miguel Angel Sebastian

**Affiliations:** 1 Departamento de Ciencias de los Materiales e Ingeniería Metalúrgica, Expresión Gráfica en la Ingeniería, Ingeniería Cartográfica, Geodesia y Fotogrametría, Ingeniería Mecánica e Ingeniería de los Procesos de Fabricación, Universidad de Valladolid, Valladolid, Spain; 2 Department of Manufacturing Engineering, Universidad Nacional de Educación a Distancia, Madrid, Spain; UC Davis School of Medicine, United States of America

## Abstract

**Background:**

Musculoskeletal disorders (MSDs) that result from poor ergonomic design are one of the occupational disorders of greatest concern in the industrial sector. A key advantage in the primary design phase is to focus on a method of assessment that detects and evaluates the potential risks experienced by the operative when faced with these types of physical injuries. The method of assessment will improve the process design identifying potential ergonomic improvements from various design alternatives or activities undertaken as part of the cycle of continuous improvement throughout the differing phases of the product life cycle.

**Methodology/Principal Findings:**

This paper presents a novel postural assessment method (NERPA) fit for product-process design, which was developed with the help of a digital human model together with a 3D CAD tool, which is widely used in the aeronautic and automotive industries. The power of 3D visualization and the possibility of studying the actual assembly sequence in a virtual environment can allow the functional performance of the parts to be addressed. Such tools can also provide us with an ergonomic workstation design, together with a competitive advantage in the assembly process.

**Conclusions:**

The method developed was used in the design of six production lines, studying 240 manual assembly operations and improving 21 of them. This study demonstrated the proposed method’s usefulness and found statistically significant differences in the evaluations of the proposed method and the widely used Rapid Upper Limb Assessment (RULA) method.

## Introduction

Human factors are strategic in the manual assembly process design. It is common to find a workstation with a design that does not adequately fulfill the ergonomic requirements for correct manual assembly. Musculoskeletal disorders (MSDs) are a result of these poor ergonomic designs and are the occupational illness of greatest concern, representing the main cause for leaves of absence from employment in Spain [1]. In 2011, the number of occupational accidents numbered 512,584, with 38.5% involving MSDs. In addition, occupational illnesses numbered 18,121, with 71% involving MSDs [2]. In this regard, efforts to ensure ergonomic optimization of the assembly line require both significant support and safer lines. Thus, human factors should be considered from the initial design phase. The number of prototype workstations to test the assembly line should be reduced, which could avoid errors resulting from machine specification and could help to eliminate installations that produce accidents or injuries.

Simulation tools are already widely used in different fields of product-process engineering, such as the study of mechanical behavior, vibro-acoustic feedback from different controls, or machine parameter estimates in manufacturing processes [3,4]. When using these tools, it is necessary to define the material characteristics and to use a good standard that determines the model that would best represent the performance of the system. From the process design perspective, decisions can be made concerning the choice of material and the machine parameters and specifications. These data are very important in the final product-process design and could be said to represent a “microscopic vision of the process”, which leads to maintaining the “good parts” and losing the “overall or macroscopic vision of the productive process”, where the final interaction between the raw products (raw material and components), the resources (machines, equipment, tools, and human factors) and the process itself (production demands, method, production mix, lay-out, and planning) results in the final product.

In an ergonomic optimization line of productive processes from the human factor perspective, Chafin (2007) [5] affirms that introducing digital human models that enable the study of product and process adaptation for people without any need of physical prototypes can reduce the development time and costs. Different applications and developments concerning the use of 3D simulation tools for the evaluation of workstations can be read in the scientific literature, successfully describing the use of these tools for the design and improvement of workstations [6–9]. In addition, different studies associate the improvement of ergonomics with quality and productivity improvement [10–13].

There is a general interest from manufacturing engineers, ergonomists, prevention specialists, operatives, union representatives, and government institutions to assess risk as the first step in the prevention and reduction of these types of injuries. Accordingly, the principles of ergonomics can be integrated with the work method design, the interaction between worker and machine in the workstation, and, in general, the overall design of the workstation where the worker utilizes this method [14]. There are ergonomic tools on the market that have been developed for different types of industries. When trying to fulfill their mission, these tools do not provide a fully satisfactory response as a MSD risk prevention tool in all fields due to a variety of factors, including high costs, the validity of results, the time used to perform the studies, the failure to implement steps, and work areas being non-compatible with existing tools. A postural assessment method for manual assembly that would reduce the likelihood of MSDs could be integrated with the existing tools already used in product-process development. This method would also work as a risk prevention tool of MSDs. In addition, this method helps to assess the workstation and quantify the improvement in ergonomic interventions in the manufacturing engineering environment.

This paper presents the development, application, and first evaluation of a postural assessment method for specific application within a manual assembly environment, allowing for the comparison of different design alternatives produced in the design phases, a detailed design, and continuous improvement of the projects. The development of the proposed method has centered on using a digital human model (DMH) integrated with a 3D product-process design environment. This Novel Ergonomic Postural Assessment Method (NERPA) approach, as a modified version of the Rapid Upper Limb Assessment (RULA) method [15], was developed for use in industrial manual assembly tasks typical in the automotive industry. Effectiveness of RULA and NERPA was compared using a real manufacturing process

## Methods

### Generation of the new method

From the risk and design prevention perspectives, as well as for assessment and continuous improvement within manufacturing engineering, the need to count on a fast, easy, and inexpensive method in the postural assessment of a workstation has led to a proliferation in the use of different observation methods. Among all of the observation methods, RULA is one of the most commonly used in industrial environments [16]. Observation methods integrated with graphic design tools in the preliminary design phases are an important element to assess work posture in a conceptual design environment. Using a DHM, the RULA method is used in different 3D graphic design environments and is able to assess worker posture and measure the level of risk.

Several studies from the scientific literature confirm that the RULA method detects risk situations if workers report discomfort but that the reverse case may not always be true [17–20]. There are examples of practicing ergonomic improvements in workstations where the assessment of these improvements using the RULA method is not reflected in a substantial reduction in risk level despite the fact that the workstation did improve [21–25]. On several occasions, ergonomic assessments using the RULA method appear very strict, whereas on other occasions, its use illustrates the difficulty of finding assessments with risk-free evaluations for workstations despite the availability of other methods for the same task [26,27].

**Figure 1:**
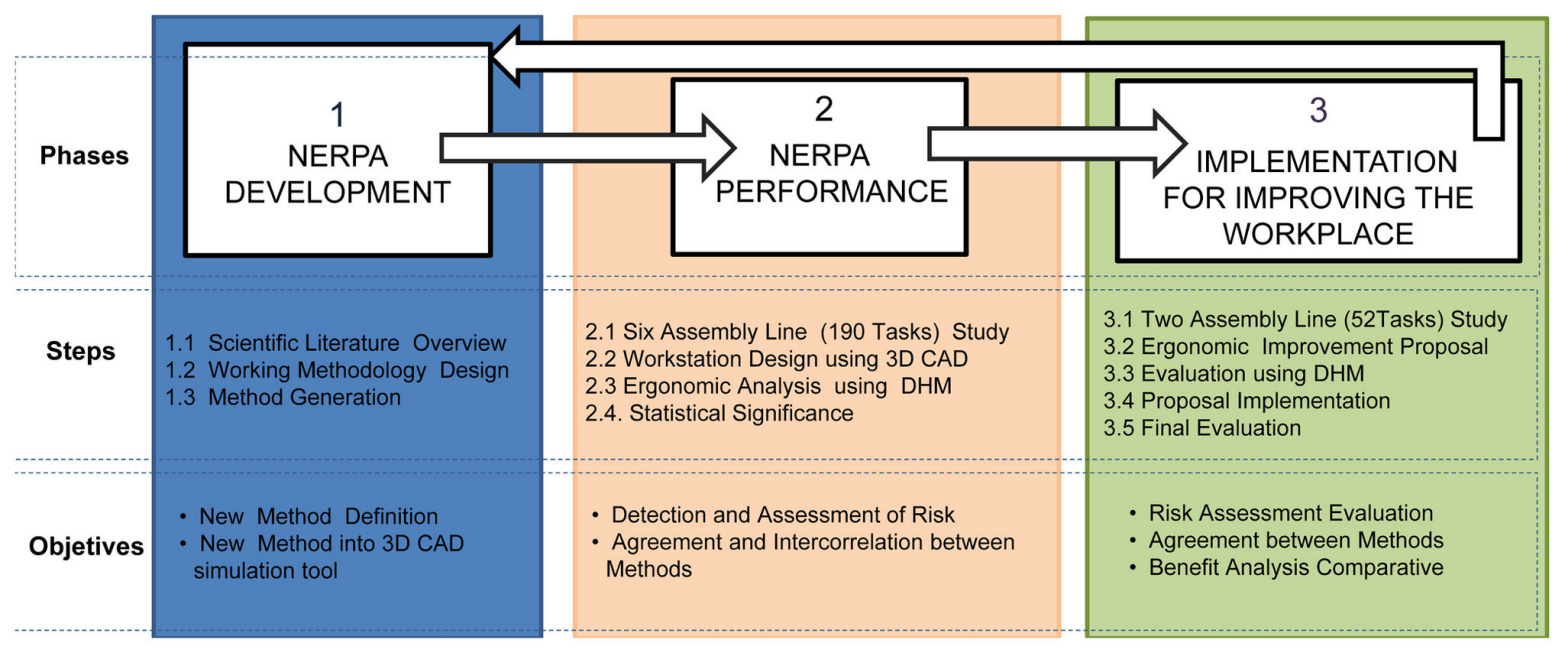
Generation of the new method: Working methodology (Phases, Steps and Objectives).

As Drinkaus et al. (2003) [28] note, the automobile industry is an excellent example of the maximization of the use of time for manual assembly. In addition, in this industry, each workstation has a wide variety of movements such that all operations undertaken in the time cycle can be divided into small tasks. When an industrial manual assembly workstation is analyzed, the worker is typically found standing in front of the transfer line. The worker does not handle or transport heavy loads and typically does not move around too much (his work area in many cases does not exceed 1.5 m). He does not remain in a static position for any significant period of time. It is mainly the upper extremities, such as the arms, trunk, neck, wrists, and hands that are involved in the movements that are performed. In this regard, although the RULA method is a good starting point to ergonomic postural assessment, this method does not present completely positive results for industrial manual assembly workstations (see Table 1) and provides a very conservative focus in the evaluation of risk [29–33]. The NERPA method has been developed to overcome these disadvantages. Figure 1 illustrates the phases, steps, and objectives of its development.

**Table 1:** RULA method for ergonomic industrial manual assembly workstation assessment: advantages and disadvantage.

**Advantages**	**Disadvantages**
Fast and easy to use	Episodic
Evaluation without performing any experimental measurements.	Difficult to reflect a safe workstation [18–23,25,32]
The result is a value, easily comparable and has an action associated with the improvement	Risk is identified with a more significant risk than they may really have [28–30]
Covers the external MSDs factors McPhee (1987) [33]	Overall indicator not efficiently allows for risk control [31]
Implementation with a computer-assisted tool is easy	RULA Validation is based on mono-task
This method is known within the automotive sector	

With the aim of modifying posture classifications and searching for observation methods and standardization, as established by Juul-Kristensen et al. (1997) [34], appropriate modifications in the corresponding scores for body parts were made. The angular values of each body group were modified, starting with the RULA method and using the standards shown in Table 2.

**Table 2:** Ergonomic standards considered in NERPA method.

**Ergonomic Standards**
ISO 11226: 2000 Ergonomics – evaluation of static working postures [39]
ISO 11226:2000/Cor 1:2006 Ergonomics evaluation of static working postures [40]
UNE-EN 1005-4+A1:2009 Safety of machinery - human physical performance - part 4: evaluation of working postures and movements in relation to machinery [41]
UNE-EN 1005-5:2007, Safety of machinery – human physical performance – part 5: risk assessment for repetitive handling at high frequency [42]

The performance of the NERPA method and its comparison with the RULA method were evaluated in six automobile manual assembly lines. A total of 190 tasks were chosen to perform postural analysis with the RULA and NERPA methods in a 3D simulation environment. Two routines were programmed to obtain the evaluation of the RULA and NERPA methods with the 3D graphic simulation tool. Different workstations were studied in each one of these lines. Each workstation had different tasks, such as part assembly, the removal of finished parts to the container, the collection of material, and the insertion of parts into machines. The ergonomic studies were performed for postures posing the greatest risks. These studies were undertaken in a 3D virtual environment that faithfully reproduced the working conditions.

**Figure 2:**
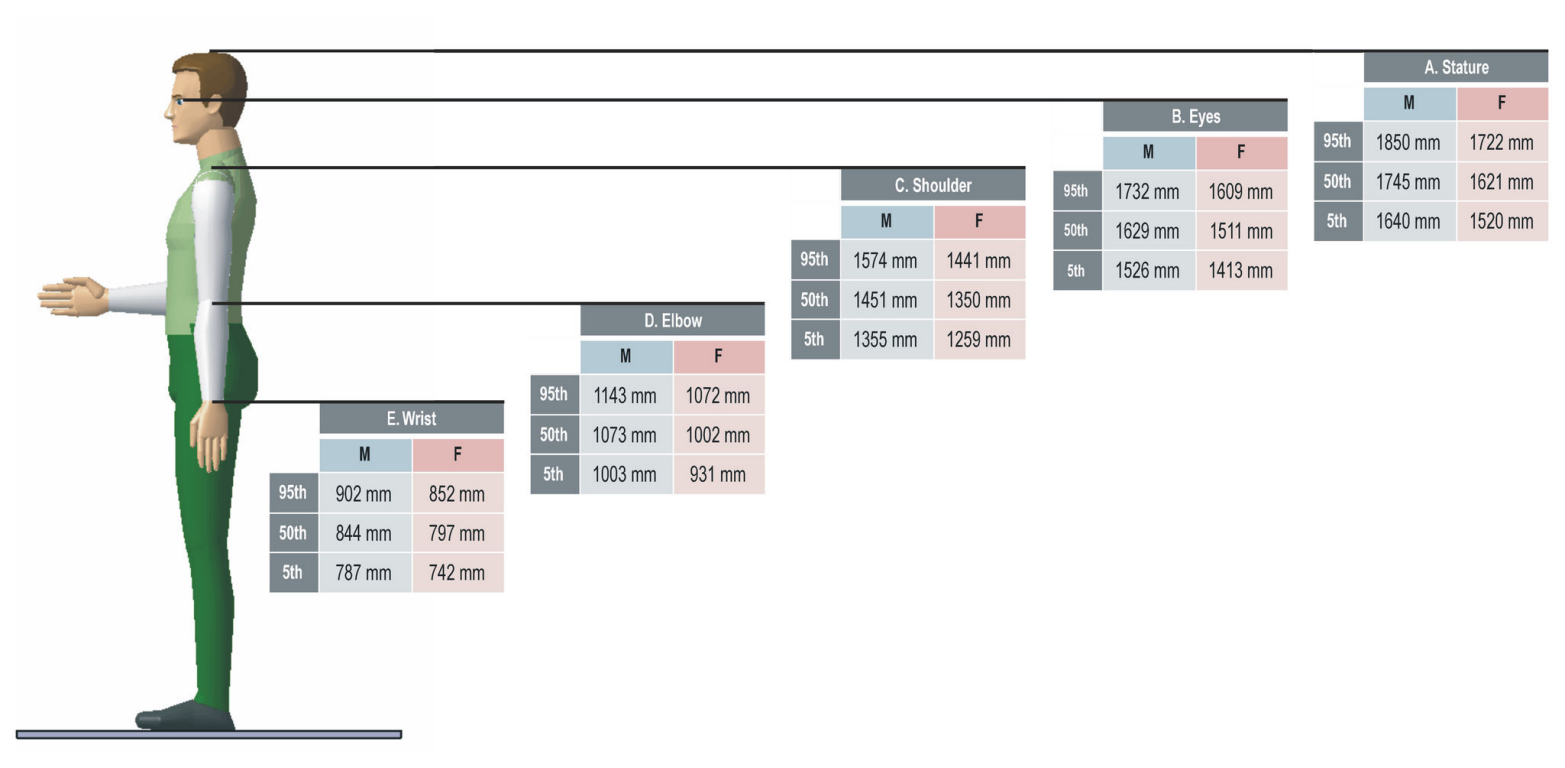
Main anthropometric dimensions A: Stature, B: Eye height; C: Midshoulder height, D: Elbow height; E: Wrist height.

To conduct the ergonomic evaluations in a simulation environment, it is necessary to define all of the resources, including the 3D geometry of the workstation, a DHM, the 3D geometry of the assembly of parts, and a definition of all assembly tasks. The 3D workstation includes equipment, shelves, containers, tools, and worktables. The DHM covers the range of the population that will perform the assembly. In our case, two percentiles that represented the lowest and highest values of the factory taskforce were used, namely, 5th percentile women and 95th percentile men. Figure 2 provides the main anthropometric values. All of these variables establish the virtual workstation where simulation and ergonomic assessment are conducted.

We applied the Kruskal-Wallis one-way analysis of variance [35] to determine whether the NERPA and RULA methods are non-related. The Kruskal-Wallis test is a non-parametric procedure that does not assume a normal distribution and allows studying groups of unequal size. To analyze the statistical significance of final RULA and NERPA scores, one sample of the final RULA scores for each workplace was used against one sample of the final NERPA scores of the same workplace. Each sample contends every final score for all evaluated tasks into one workplace (for number of task, see table 3 column 2). The proposal Kruskal-Wallis test for two samples is fundamentally the same as Mann-Whitney U test (Wilcoxon test). Both methods have identical mathematical p-value. Final RULA and NERPA scores were ranked prior to the analyses. Also we applied the Kruskal-Wallis one-way analysis of variance take into account all the workplaces. This test contends two samples, and each sample contends every final score for all evaluated tasks into all workplace (190 tasks). We considered p-values of 0.05 or less to be statistically significant. The statistical analysis was performed using G-Stat Statistical Software [36].

**Table 3:** Agreement between final RULA and NERPA evaluations, and Kruskal-Wallis test p-value and degree of freedom (DF) (final score H means high risk, M moderate risk and L slight or low risk).

**Line Number**	**Total Tasks**	**Time (s)**	**R(M)/N(L)**	**R(M)/N(M)**	**R(H)/N(L)**	**R(H)/N(M)**	**R(H)/N(H)**	**p-value**	**DF**
FMA_001	28.00	36.00	14.3%	64.3%	-	21.4%	-	0.0014	1
FMA_002	30.00	45.00	33.3%	46.7%	-	20.0%	-	0.0052	1
FMA_003	38.00	38.00	-	63.2%	-	26.3%	10.5%	0.0190	1
FMA_004	44.00	57.00	45.5%	22.7%	-	18.2%	13.6%	0.0040	1
FMA_005	20.00	62.00	-	30.0%	-	40.0%	30.0%	0.0380	1
FMA_006	30.00	55.00	33.3%	46.7%	-	20.0%	-	0.0250	1
All	190.00		24.4%	43.6%	-	23.4%	8.5%	0.0001	1

### Implementation of the new method for workplace improvement

**Figure 3:**
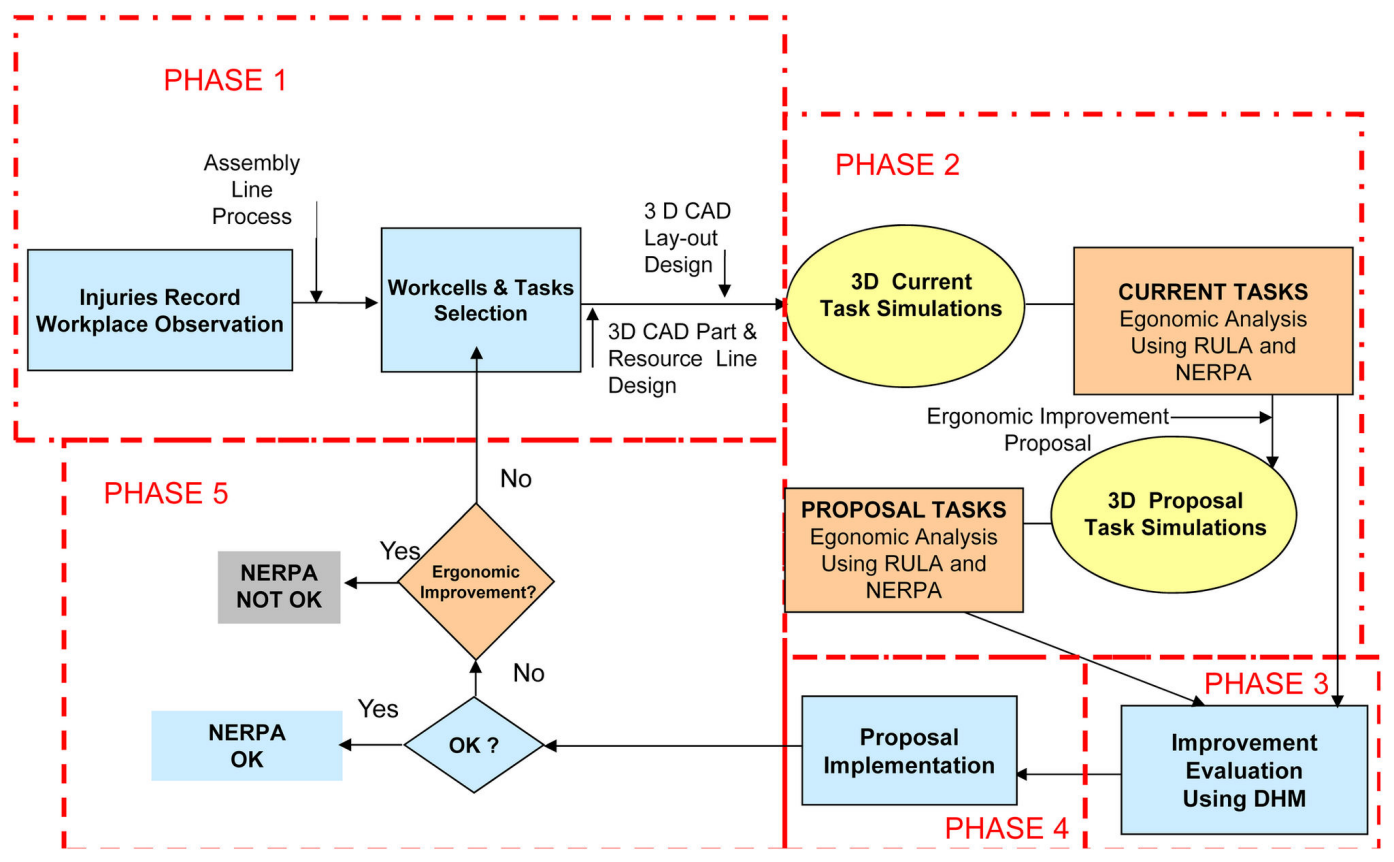
Ergonomic workplace improvement using NERPA Method: Methodological Approach.

The ability of the NERPA method to detect improvements in the workstation was evaluated in the final stage of method development. Figure 3 illustrates the steps used for the methodological approach.

**Figure 4:**
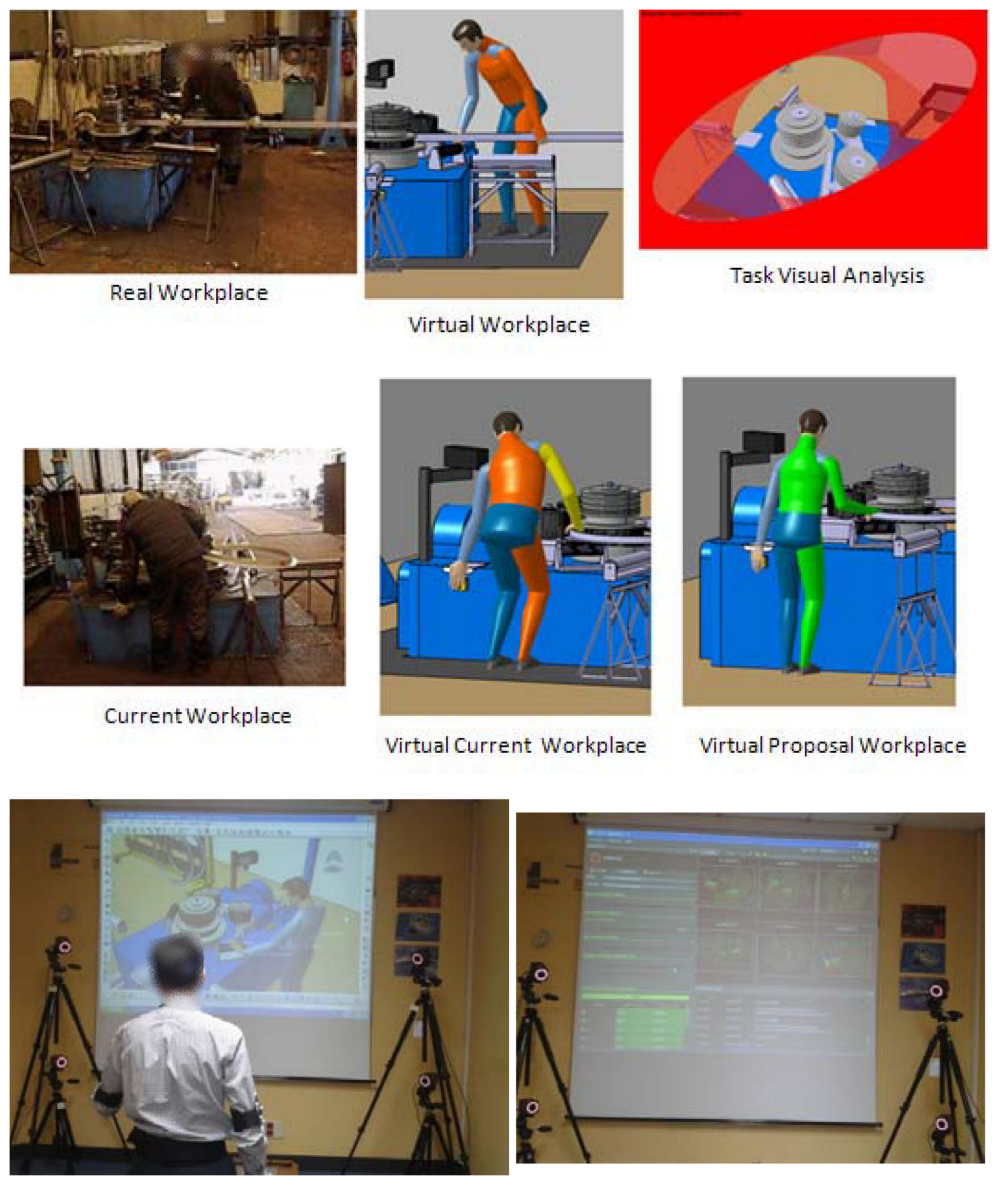
Real task, virtual task ergonomic evaluation and Motion Capture System laboratory workplace.

Worker opinions and the record of injuries were collected in phase 1, choosing a total of 26 tasks. In phase 2, postural analysis was performed using the RULA and NERPA methods in all tasks. Analysis was performed again in a 3D simulation environment, with its own implementation of the RULA and NERPA assessment methods. They were presented in the same manner as in the previous section. The improvement alternatives were presented in the 3D simulation environment. In phase 3, ergonomic improvements were assessed in the conceptual environment stated in the previous phase. In the next phase (phase 4), an optical Vicon’s tracking system composed of six infrared Bonita cameras integrated in the 3D simulation design environment was used to evaluate the alternatives (see Figure 4). This real time 3D optical tracking system has 0.1 mm positional and 0.15^°^ angular accuracy average [37]. The system was implemented for laboratory workplace ergonomic evaluation and to gather worker posture when carrying out the operations before implementation in the real workplace. Nordic questionnaire [38] was used in order to gather the worker opinion. Posture was recorded helping by tracking system. All postures were evaluated into de 3D real time simulation environment. Worker opinion, RULA and NERPA final scores were compared before and after proposal implantation. The best ergonomic proposals were included in the real workstations. Finally, in phase 5, improvements were evaluated in the real workstations. NERPA was tested considering the record of injuries before and after the proposal implementation and the assessment matrix of Table 4.

**Table 4:** Matrix NERPA workstation assessment versus Real workstation after improvement have been established.

**NERPA WORKSTATION ASSESSMENT**	**REAL WORKSTATION Ergonomic Improvement**	
**Ergonomic Improvement**	**YES**	**NO**
**YES**	NERPA OK	
**NO**		NERPA OK

## Results

### NERPA assessment worksheet

The three main results of the study will be discussed in this section. They can be summarized as the NERPA assessment worksheet, NERPA performance, and NERPA benefit analysis.

**Figure 5:**
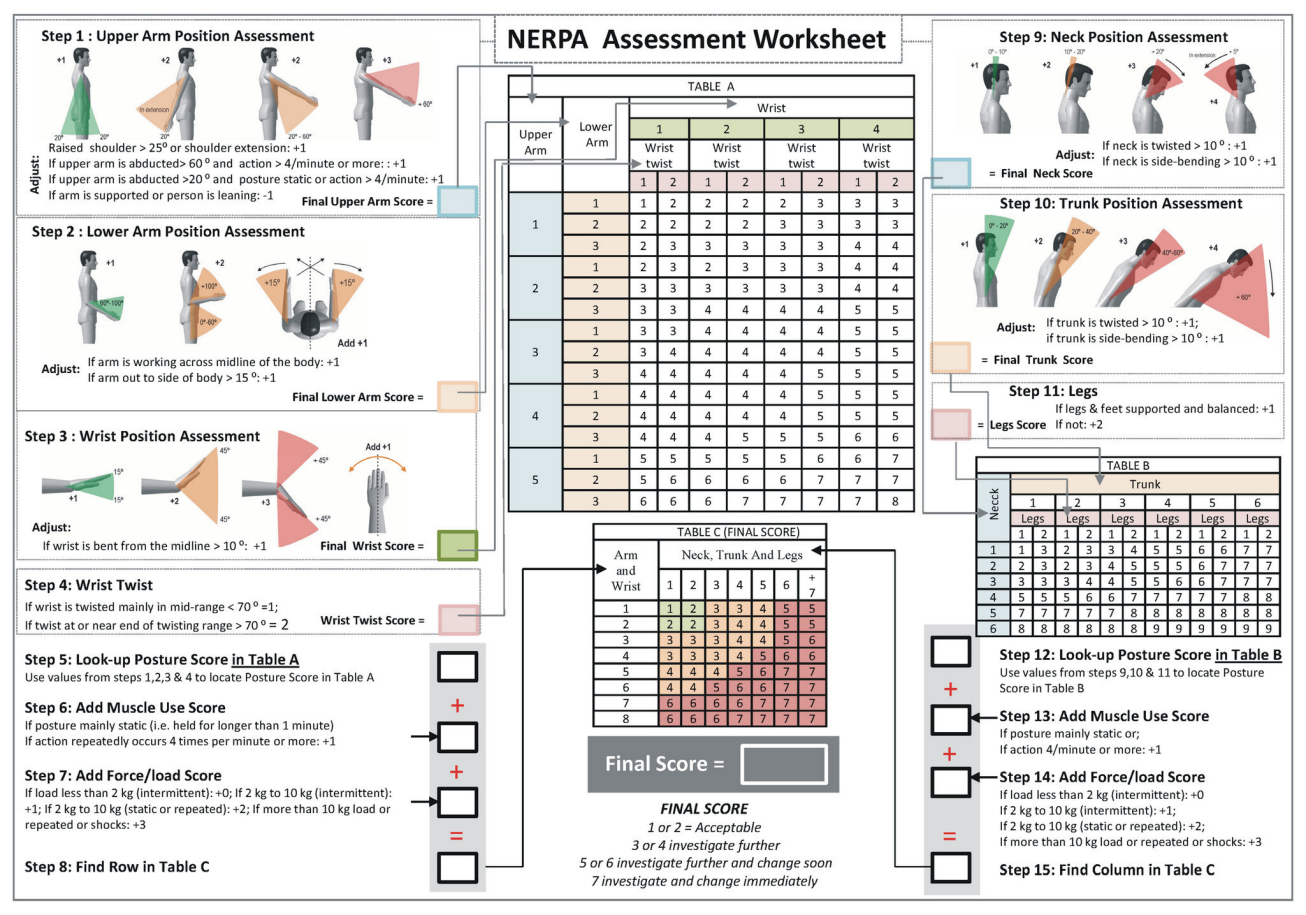
NERPA worksheet modified from RULA worksheet using new NERPA criteria.

The NERPA assessment worksheet is shown in Figure 5. This worksheet attempts to explain the NERPA method in great detail by showing every step to complete an ergonomic task assessment. The approach of the new method begins with the premise of maintaining the original A, B, and C tables of the RULA method. In this manner, the final results of the method may be identifiable with the RULA method, facilitating the acceptance and understanding of the results in areas of manufacturing where RULA has already been used. The NERPA method does not use modifications to assess the legs but presents changes for the arms, neck, trunk, and wrists. Following this reasoning, the performance in every part of the body with modified scores is shown.

#### New upper arm assessment with the NERPA method

Four positions are considered for bending the arm in the RULA method. Standard ISO 11226:2000 [39,40] establishes three ranges of scores instead of four. These three ranges were used in the new method for this segment. The range of movement is expanded in this manner and does not penalize those common work positions that do not constitute, a priori, any risk for the worker.

The first level in the NERPA method remains the same as in RULA. The second level increases by 15^°^ to achieve greater flexibility. However, the third level decreases by 30^°^ (a 90^°^ limit becomes 60^°^), and the fourth level is eliminated because the vast majority of the postures do not require a total flexion of the arm. The movement of the trunk complements such movements.

Standards UNE-EN 1005-4+A1:2009 [41], UNE-EN 1005-5:2007 [42] and the OCRA method [43] were adopted for the rest of the joint values (see Figure 5). As shown in Figure 5, the NERPA method offers a variety of four possibilities to choose the addition of the value (+1/-1). The factor for postures in favor of gravity remained unchanged.

#### New trunk assessment with the NERPA method

In NERPA, when considering the trunk inclination movement, the first score is increased by 10^°^ and the second by 20^°^ compared to RULA, and in the third score, the upper limit remains the same, whereas the lower limit is increased by 10^°^. These three modified scores yield movement values that are more suitable for the work activity. To establish the first level of penalization (0-20^°^ of flexion), the values were obtained from Standard ISO 11226:2000. Similar to the flexion of the trunk, the angular values corresponding to twisting and lateral inclination were obtained from Standard ISO 11226:2000.

#### New neck assessment with the NERPA method

In the RULA method, if the neck suffers torsion or inclination, this movement is penalized with a +1 factor, without any other consideration, which is very strict and does not reflect an adequate assessment of the movement. There is a certain margin to be considered in the NERPA method before penalizing the movement. In this manner, if the neck experiences torsion or inclination higher than 10^°^, +1 must be added to obtain the final neck score. However, the neck flexion values remain the same as in RULA.

#### New wrist assessment with the NERPA method

The RULA method is overly restrictive regarding the flexion of the wrist. It does not allow for any range of movement of the wrist without penalization. This restriction is excessive because it is necessary to bend the wrist at least slightly in almost every workplace. Thus, in NERPA, a small margin is given for this movement (15^°^) without penalization. This margin is based on the OCRA method strategy.

As in almost all other body segments, factors for other possible movements associated with the wrist must be considered. Accounting for the criteria of Weiss et al. (1995) [44] and Werner et al. (1997) [45], which indicate that an increase in the risk of injury exists if a flexion higher than 30^°^ and a radial deviation higher than 10^°^ occur simultaneously, a penalty occurs under a radial deviation higher than 10^°^ or a cubital deviation lower than 10^°^.

The RULA method does not provide exact values for the wrist twist. RULA only considers joints of medium and extreme degrees. Given the limit for the rotation of this joint [46], 70^°^ has been estimated as the limit value.

### NERPA performance

#### Detection, risk assessment, and agreement between methods

A total of 190 tasks were studied related to manual operations for transportation, material supply, material guidance to machine, the capture of parts for assembly from different containers at the line level, machine handling, and the removal of finished parts to containers. Given the great capacity of the tools, not only were posture and accessibility evaluated but also visibility when performing the task (see Figure 4).

**Figure 6:**
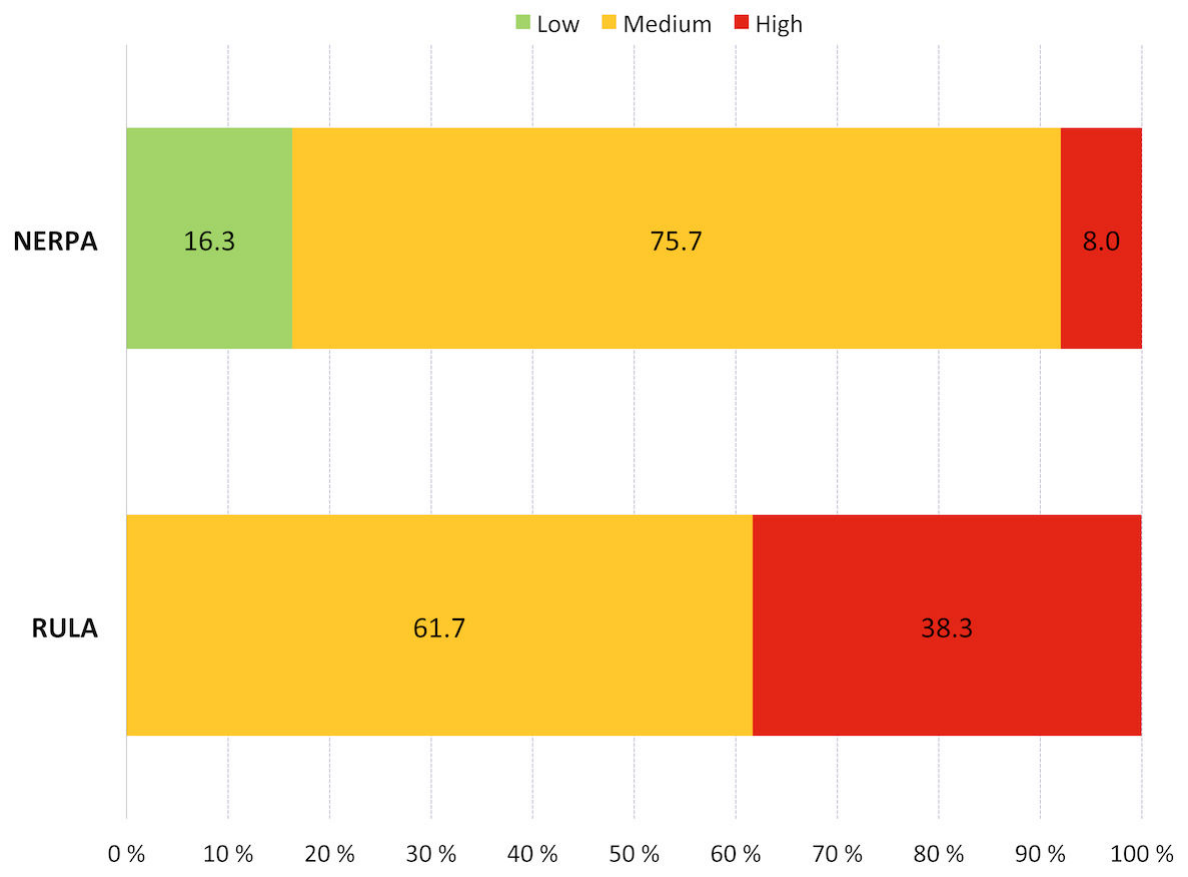
Ergonomic Evaluation using NERPA and RULA.

To compare the methods, the final assessments were divided into three groups: *low risk* (L), represented in green with scores from one to two; *medium risk* (M), in orange with scores from three to four, and *high risk* (H), represented in red with scores from five to eight, as shown in Figure 6. A comparison of the evaluations obtained indicates that both methods are capable of detecting postures with ergonomic risk. However, the RULA method is not able to recognize operations without risk. NERPA indicates 16.3% of operations that can be considered injury free.

#### Agreement and inter-correlation between methods

In accordance with the established classifications in the groups of the final assessments (L, M, H) explained in the previous section, Table 3 summarizes the degree of agreement and disagreement between both methods. The results obtained by both methods for each operation studied in each assembly line do not coincide by more than 52%. This percentage is low if we consider all of the operations studied regardless of the line to which they belong.

The Kruskal–Wallis test was used to find significant differences between the two methods. The greatest p-value found was 0.038 (see Table 3); p-values of 0.05 or less were considered statistically significant, concluding that the assessments of the two methods lead to significantly different results. NERPA and RULA are not related.

### NERPA benefit analysis

After the assessment, several improvement proposals were developed that modify the altimetry of the machine, the equipment design for the introduction of material, the removal of finished parts, and the reorganization of containers and shelves at the line level. An analysis of the values assigned by RULA at the workstation including the improvement proposals indicates a decrease in scores in general. This method only detects one task without risk after the improvement proposals have been applied. The remaining proposals did not succeed in reducing or avoiding risks at workstations. However, NERPA reduces the scores and action levels, which helps to detect more proposal improvements that help to identify more low-risk operations.

Table 3 illustrates how the RULA and NERPA methods detect risk after the implementation of proposals. If we analyze the results, several proposals can lead to safer workstations characterized with low risk according to NERPA. In contrast, RULA would characterize these workstations with medium risk. Medium and high risks after the implementation of the proposals are presented in similar percentages by both methods. Tables 5-7 demonstrate that the implementation of the NERPA method allows for an improvement of nearly 16% more than that obtained with the RULA method. The number of improvement proposals that are considered safe following the RULA method is 40% lower than that following the NERPA method (see Tables 6 and 7). Thus, the RULA method could represent a loss of opportunity.

**Table 5:** Agreement between final RULA and NERPA evaluations at workplace improvement.

	**Total Tasks**	**R(L)/N(L)**	**R(M)/N(L)**	**R(M)/N(M)**	**R(H)/N(L)**	**R(H)/N(M)**	**R(H)/N(H)**
Current Tasks	25.00			32.00%		4.00%	52.00%
Proposal Tasks	25.00		68.00%	16.00%		16.00%	

**Table 6:** Current vs proposal tasks: RULA analysis.

**RULA PROPOSAL TASKS**		**RULA CURRENT TASKS**	
TASKS	**LOW**	**MEDIUM**	**HIGH**
**LOW**		12%	20%
**MEDIUM**		20%	29%
**HIGH**			16%
**RULA Global Improvement**			**61%**

**Table 7:** Current vs proposal tasks: NERPA analysis.

**NERPA PROPOSAL TASKS**	**NERPA CURRENT TASKS**		
	**LOW**	**MEDIUM**	**HIGH**
**LOW**		40%	32%
**MEDIUM**		12%	5%
**HIGH**			
**NERPA Global Improvement**			**77%**

Finally, improvements were evaluated in the actual workstations, detecting a 43% decrease in the number of injuries on the line following the improvement proposals evaluated using the NERPA method. Number of injuries was evaluated in eighteen months.

## Discussion

Tables 8-12 offer a comparison between the values of joint angles of the NERPA, RULA, OCRA, and UNE-EN 1005 for the wrist, arm, forearm, back, and neck. To compare the ranges of evaluation according to the angles of each method, within each body part, each angular assessment is divided into three sections (similar to the OCRA method). These sections identify the absence of risk or possible low risk (green), moderate risk (orange), and high risk (red). The parts evaluated with four sections in the RULA method were grouped in three sections. These tables indicate that in general, the NERPA values are less restrictive than those obtained with the RULA method.

**Table 8:** Wrist Movements: Comparison between the values of joint angles of NERPA, RULA, OCRA, and UNE-EN 1005.

**Movement**	**Range**	**RULA**	**NERPA**	**OCRA**	**1005-4**	**1005-5**
**Flexion**	Green	0	0 - 15	0 - 45	-	0 - 45
	Orange	0 - 15	15 - 45	> 45	-	> 45
	Red	> 15	> 45	> 45	-	> 45
**Extension**	Green	0	0 - 15	0 - 45	-	0 - 45
	Orange	0 - 15	15 - 45	> 45	-	> 45
	Red	> 15	> 45	> 45	-	> 45
**Radial Deviation**	Green	0	0 - 10	0 - 15	-	0 - 15
	Orange	0	> 10	> 15	-	> 15
	Red	> 0	> 10	> 15	-	> 15
**Ulnar Deviation**	Green	0	0 - 10	0 - 20	-	**0 - 20**
	Orange	0	> 10	> 20	-	**> 20**
	Red	> 0	> 10	> 20	-	**> 20**

**Table 9:** Lower Arm Movements: Comparison between the values of joint angles of NERPA, RULA, OCRA, and UNE-EN 1005.

**Movement**	**Range**	**RULA**	**NERPA**	**OCRA**	**1005-4**	**1005-5**
**Flexion - Extension**	Green	0 - 20	0 - 20	0 - 20	**0 - 20**	< 80°
	Orange	-20-0;20-45	20 - 60	20 - 60	**20 - 60**	90% time
	Red	>45 ;> 90	> 60	> 60	**> 60**	
**Abduction - Adduction**	Green	0	0 - 20	0 - 20	**0 - 20**	90% time
	Orange	> 0	20 - 60	20 - 60	**20 - 60**	
	Red	> 0	>60	> 60	**> 60**	
**Rotational**	Green	-		-	**-**	
	Orange	-		-	**-**	
	Red	> 0	>15 >60	-	**-**	

**Table 10:** Upper Arm Movements: Comparison between the values of joint angles of NERPA, RULA, OCRA, and UNE-EN 1005.

**Movement**	**Range**	**RULA**	**NERPA**	**OCRA**	**1005-4**	**1005-5**
**Flexion - Extension**	Green	0 - 20	0 - 20	0 - 20	**0 - 20**	< 80°
	Orange	-20-0;20-45	20 - 60	20 - 60	**20 - 60**	90% time
	Red	>45 ;> 90	> 60	> 60	**> 60**	
**Abduction - Adduction**	Green	0	0 - 20	0 - 20	**0 - 20**	90% time
	Orange	> 0	20 - 60	20 - 60	**20 - 60**	
	Red	> 0	>60	> 60	**> 60**	
**Rotational**	Green	-		-	**-**	
	Orange	-		-	**-**	
	Red	> 0	>15 >60	-	**-**	

**Table 11:** Trunk Movements: Comparison between the values of joint angles of NERPA, RULA, OCRA, and UNE-EN 1005.

**Movement**	**Range**	**RULA**	**NERPA**	**OCRA**	**1005-4**	**1005-5**
**Flexion - Extension**	Green	0 - 20	0 - 20	-	**0 - 20**	-
	Orange	20-60	20-60	-	**20-60**	-
	Red	< 0 ; > 60	0 <> 60	-	**0 ; > 60**	-
**Lateral Bend**	Green	0	0-10	-	**0-10**	-
	Orange	> 0	-	-	**-**	-
	Red	> 0	> 10	-	**> 10**	-
**Upward- Downward Rotation**	Green	0	0-10	-	**0-10**	-
	Orange	> 0	-	-	**-**	-
	Red	> 0	> 10	-	**> 10**	-

**Table 12:** Neck Movements: Comparison between the values of joint angles of NERPA, RULA, OCRA, and UNE-EN 1005.

**Movement**	**Range**	**RULA**	**NERPA**	**OCRA**	**1005-4**	**1005-5**
**Flexion - Extension**	Green	0-10	0-10; 0-5	-	0-40	
	Orange	10-20	10-20	-	-	-
	Red	> 20	> 20; >5	-	> 40	-
**Lateral Bend**	Green	0	-	-	-20	
	Orange		-	-	-	-
	Red	> 0	-10 <>10	-	**-10 <>10**	-
**Rotational**	Green	0	-	-	-90	
	Orange		-	-	-	-
	Red	> 0	-10 <>10	-	-45 <> 45	-

However, as mentioned above, the validation of the RULA method is based on mono-task operations. This fact is not a problem in the field of action of this paper but is instead an advantage. It may be possible for the operator to recover between one operation and the next. He/she is able to adopt different postures and thus be somewhat conservative in the development of the new method and even be flexible with the postural values of the new criteria. Observing the results of NERPA in the first assessment, previous to the proposals, it is clear that the percentage of cases that do not need to be studied for improvement is greater than with RULA. Moreover, when considering the results of NERPA after the application of the proposals, the number of cases to be restudied is less than in the RULA method, which decreases the costs of rethinking, reengineering, and reworking as well as the resulting investments.

The second determining factor of the tool was its capability of providing a quick assessment. NERPA could be used in a 3D CAD environment, but the manufacturing engineering of the factory could also use it to assess its workstations in an acceptable time period and without making significant investments. Ergonomic Postural assessment using 3D CAD lets postural assessment easier, but 3D CAD tool user knowledge is an important factor. In this respect, a correlation between an experimental test (using goniometric measurements for upper limbs) and 3D CAD simulation for simple movements gave differences less than 5^°^ for angular values. Using a Vicon’s real time tracking system into a 3D CAD simulation reduces this value.

The new method has demonstrated its validity using assessment and ergonomic process improvement in a real industrial environment, reducing the record of injuries caused by MSDs. However, the method must be applied to other industrial areas to obtain a more robust assessment of its capabilities.

## Conclusion

A new predictive method (NERPA) has been developed using a modified RULA method approach to be used in industrial manual assembly operations. NERPA allows the engineer to make adequate decisions in the design and postural assessment of workstations to reduce the possible risk of experiencing musculoskeletal injuries in manual assembly operations.

The method to assess postures has been developed through the use of CAD design tools and a 3D biomechanical model included in a DHM, together with the use of a system for motion capture in real time, which is used within the 3D virtual environment, allowing for the integration of the work process, resources (equipment, machine, tools), and human factors. The new method implemented in a 3D simulation tool allows for the elimination of the observation factor, the advantage of which is twofold. First, the possible error in observing the angles is avoided because the software itself provides this information (it not only provides angles but also evaluates the posture). However, because the program permits the assessment of all postures, it is easier to determine the most injurious posture.

The NERPA method, which modifies the assessment of some joint ranges while maintaining the same assessment structure as the RULA method, presents significant differences with respect to RULA. For the work conditions under which it was used, this method is capable of detecting postures with ergonomic risk and is more sensitive to the detection of an ergonomic improvement than the RULA method. The two methods lead to significantly different results. Under the methodological concept presented in this paper, other factors of ergonomic risk could be added to the NERPA method, which would allow for the development of a methodology of overall risk assessment for industrial production in the framework of risk prevention.
